# Social Sciences Research on Infectious Diseases of Poverty: Too Little and Too Late?

**DOI:** 10.1371/journal.pntd.0002803

**Published:** 2014-06-12

**Authors:** José Azoh Barry

**Affiliations:** Investigación & Acción, A.C., General Escobedo, Nuevo León, Mexico; University of Houston, United States of America

## Introduction

Infectious diseases of poverty, also labeled tropical diseases or neglected tropical diseases (NTDs) and caused by pathogenic agents (viruses, bacteria, fungi, and other parasites), are viciously more prevalent among poor people. Though being preventable for the most part in a cost-effective way, they are devastating. These are, to name a few, Chagas disease, schistosomiasis, malaria, leprosy, visceral leishmaniasis, lymphatic filariasis, Buruli ulcer, and onchocerciasis. Besides the vicious circle these diseases maintain with dire conditions of poverty, an increased microbial resistance to some therapeutic drugs adds to the complexity of health disparities and human suffering among the socially disadvantaged, marginalized, and prejudiced against. Fostering virtuous circles (as opposed to vicious circles) against infections of poverty and putting the disenfranchised first are primary concerns for social scientists engaged with research into infectious diseases of poverty. The historical role of social science research into these diseases, its current impacts, substantial contributions, and opportunities and interests for future endeavors are the focus of this article. Persistent disruptions and their propensity to wholly hamper productivity, derail economic and social progress, and deny child development are part of the complex reality to look into. In forcing the displacement of populations and creating chaos, they increase the risk for the spread of infections and maintain the infected poor in a downward spiral of poverty through their capacity of securing the vicious relationship with NTDs. Rather than compassion for inequalities, vulnerabilities, deprivations and misery, or bad fate, foci such as social justice, preparedness, and empowerment are of utmost importance. The case for bridging the divide among scientific disciplines has been strongly made over the years by scholars and outside of academic institutions. Acknowledging the importance of interdisciplinary science and contemplating the need for funded multidisciplinary research is hopeful for broadening the expertise needed to tackle these multidimensional afflictions. However, it should also call for a cautious enthusiasm.

## Background

Infectious diseases of poverty as a whole are widespread [1], are characterized by high morbidity and mortality rates [2], and have a high prevalence among poor people. These diseases and poverty are trapped in a sort of permanent relationship of a double layer of infections and poverty, in which pathogens infect people living in persistent conditions of poverty, and the infected poor people are ceaselessly poor and infected. Many of these pathologies have been referred to as NTDs for diverse reasons. They have, for the most part, received lesser investment (note 1 in Text S1) in terms of proper attention and actions, or have simply been ignored by the afflicted communities, media, governments, research funders, the pharmaceutical industry, and the rest of the world [3]–[5].

Breaking the vicious circle of poverty and infectious diseases and reaching disenfranchised populations are two of the compelling reasons for research into infectious diseases of poverty [2]. In accordance with the endeavors of the social sciences in this field, these reasons suggest opening new windows of insights, partnerships, reviewed initiatives, and updated research foci to care for opportune research on NTDs, rather than perpetuating its neglect. However, critical lens based on comparing performance metrics in the biomedical research and the social sciences research on NTDs may raise eyebrows.

Key findings derived from a Scopus database search are provided in a bibliographic analysis that is part of a series and considered the publications on four of these diseases over a period of ten years. They are summarized as follows: (a) research identified as social sciences represented about 2.1% of the total output with, respectively, dengue (n = 2,344), visceral leishmaniasis (n = 1,648), onchocerciasis (n = 483), and chikungunya (n = 274); (b) departments associated with strong clinical and biomedical disciplines are surprisingly productive in social sciences papers [6]. To quote these authors in their conclusion: “The evidence from the literature, however, is that there is little investigator-driven social science to speak of in the NTDs, and a similarly poor presence of interdisciplinary science. Without this, our understanding and management of NTDs is inevitably reduced to a strategy that relies on a repetitive, reductionist, flat-world science to overcome an acknowledged complex system.”

Should these statistics lead to the stigmatization of social scientists as underachievers in a particular research field? In confronting the evidence of a lower productivity reflected in counting publications, should a major focus on “n,” the absolute number, matter more than policy impact?

The case is not made for praise, yet the highlighted discrepancy may tend to overlook the historical role of the social sciences in research on NTDs. And from such a stance, this viewpoint article seeks to briefly point out the importance of depth and significance of research impacts on the targets. Rather than a list of priorities, it aims to offer some comments about current and future research into infectious diseases of poverty. The terms “infectious diseases of poverty,” “infections of poverty,” and “NTDs” are used interchangeably.

## Discussion

Translation of research into policy and a greater attention to grassroots demand in resource-poor areas are evident in social-sciences–related research and innovation applied to HIV/AIDS and Chagas disease, respectively in some African and South American countries. For those living in conditions of poverty, and especially when those conditions are extreme as it is the case in South Africa, local mobilization to speak out against HIV/AIDS appeared crucial to demanding antiretroviral therapy. Comprehensive research on Chagas disease [7], not exempt from criticism for its shortcomings [8], stood out as best practice in improved housing against infestation by *Trypanosoma cruzi* (via the *vinchucas*) [9] and became a classic. Similar work [10] contributed to such perspective on the strong relationships among health, disease, and economic and sociocultural contexts in addressing the spread of Chagas disease by triatomine vectors (*Triatoma infestans*) and the parasite (*T. cruzi*). The resulting success at inspiring sustainable, culturally sensitive and transferable community health development projects benefited the poor (Table 1).

**Table 1:** An illustration of social scientists' work on Chagas disease in South America.

Authors	Bastien, J. W.	Briceño-León, R.
**Description**		
Context	Bolivia	Venezuela
Perspective/approach	Comprehensive.	Comprehensive.
	Highlights the strong relationships among health, disease, and sociocultural contexts in the spread of Chagas disease by triatomine vectors (*Triatoma infestans*) and the parasite (*Trypanosoma cruzi*).	Highlights the precariousness of housing due to conditions of survival that deny to rural workers and their families opportunities conducive to human progress.
Features/contribution	Not exempt from criticism, but became a classic book.	Thorough analysis focused on rural poverty in Venezuela.
	Succeeded at inspiring sustainable, culturally sensitive and transferable community health development projects.	
Policy impact	Translated into policy in some South American countries (e.g., Peru, Bolivia, and Chile)	Synergistic effects of education, organizational capacity, a feeling of self-confidence, and behavioral changes.
	Best practice in improved housing against infestation by *T. cruzi* (via *vinchucas*).	

Source: Author's own elaboration.

These are illustrations of concerns for the poor, their suffering and needs, beyond generic community participation and with a further integration in medical and public-health–related interventions, by taking into account their voices in what is done in their best interest. These kinds of orientations are likely to discourage the formation of biased and well-ingrained views [11], [12] leading to negative feelings toward the poor and blaming discourses against them [13], [14].

Gender, another social aspect of NTDs, has not been neglected in research. With the implementation of the strategy to eliminate onchocerciasis, some of the sociocultural dimensions of health related to gender differences, and observed in adherence and community participation, surfaced in three African countries: Cameroon, Nigeria, and Tanzania [15]. Community-directed treatment of infectious diseases of poverty furthers the opportunity to target and act upon gender issues already identified in research conducted in Africa, Asia, and the Americas on infectious diseases of poverty. These are, for example, differentiated social stigma, economic- and social-related concerns, disease severity, and reaction to treatment in leprosy, trypanosomiasis, onchocerciasis, filariasis, and other skin-damaging and deforming outcomes [16]–[19]. Investigating the impacts of stigma on the social burden of NTDs has contributed to the assessment of the culture-specific meaning of these diseases, the evolution of the concepts of stigma, and the research and intervention interests to public and international health [20], [21].

### Infectious diseases of poverty in contemporary context

Most NTDs are found in sub-Saharan Africa, a geographical area where child poverty is still more reluctant to show a marked decline. Looking at the whole picture cannot be taken for granted, whilst disruptions and a rights-based approach to health and well-being remain overlooked pieces of the tapestry. Unnecessary and preventable armed conflicts have been linked to the spread of infectious diseases among displaced persons and refugees [22]. The resurgence of poliomyelitis, along with the impact of food- and water-borne diseases (diarrhea, cholera) and malaria on infant morbidity and mortality in Syria, South Sudan, and Central African Republic, are, too, a recent reality reported from the fields by aid agencies involved in humanitarian crisis. Less fortunate children unable to reach overwhelmed hospitals and overcrowded camps [23], or forcibly separated from their parents and at risk for being mutilated or beheaded, experience worse survival conditions hiding in the bush and swamp areas where tsetse fly, sandflies, and other vectors dwell.

Progress achieved in improving health systems and minimizing malnutrition-related problems after so many years of huge efforts, is almost wiped out within a few months or even days by political instability, unrest, and related chaos. The losses and damages caused in total violation of children's rights ensure the viciousness of NTDs, and thus, contribute to maintaining these children in a downward spiral of poverty. More than diplomacy, it is a human development and social justice issue social science research must engage with, since NTDs are also apprehended in association with the notions of vulnerability and powerlessness of their primary victims.

### Good news amidst persistent concerns

There has been good news about the trials of new pharmaceuticals and simultaneous treatments of infectious diseases of poverty. Generosity has been expressed through the free provision of the drug Ivermectin to onchocerciasis-endemic countries worldwide, and the same goes for the donation of praziquantel, a drug effective in treating schistosomiasis [24], [25]. Such progress as the use of nanomedicine brings the hope that most of the NTDs are likely to acquire the status of smallpox in the coming decades. For that to happen, universal accessibility, as an important concern, must be a high priority in research and action agendas.

Recent news about the development and clinical trials of a safe and effective vaccine against dengue are encouraging [26], [27]. Yet, once again, some crucial issues are still pending. One of these issues is the capacity of the poor who live in endemic areas to access the vaccine once licensed. Is vaccine accessibility ensured for children, the most vulnerable among the poor? Another point is that vaccine availability may not have the same meaning to the socially disadvantaged in comparison with international travelers from nonendemic countries. In addition, the latter do not confront in the same way the reality of housing standards (lack of doors and window screens, air conditioning), household food and financial insecurities (food scarcity, inability to cope with emergencies, zero savings), overcrowding (overpopulated bedrooms), viremia, and the threat these may pose to human health.

In many households from poor-resource settings, handling febrile illnesses, pediatric or not, still represents a nightmare, because the poor are more often struggling to ensure their basic living necessities. Environmental degradation, the shrinking of employment opportunities for low-skilled workers, and the decline of wages and living standards constrains their priorities and options. Difficult or unequal access to prophylactic or curative treatment, along with the use of medicinal herbs and fake pharmaceutical drugs (note 2 in Text S1) as alternatives, should be of further interest to social science research.

There are behavioral aspects related to the resistance of antibiotics, and the occurrence of infestations and reinfestations of human bodies after deworming as an effect of pharmaceuticals may be blurred by a tendency to totally rely on their efficacy. Negative health outcomes associated with unhygienic practices despite health education and promotion efforts and the implementation of improved sanitation suggest a gap in their integration into local lifestyles. Besides showing the limitations of a single approach, they also suggest a need to strike a balance as far as building partnerships with communities and owning health initiatives are concerned.

### On “the poor presence of interdisciplinary science”

Interdisciplinary science is not unknown to the social sciences. Medical anthropology itself is an interdisciplinary subfield of anthropology. Bridging the divide epidemiology and medical anthropology created by an opposition of reductionist and positivist versus humanistic and holistic was the basis for calling for the establishment of interdisciplinary linkages [28]. Looking at funded collaborative research, more predominant in the natural and biomedical sciences (note 3 in Text S1) and said to offer interesting advantages, could contribute to improve the so-called “the poor presence of interdisciplinary science.” It increases the number of articles produced with an eminent first author and published in a prestigious journal. And when collaboration is international, there is a positive correlation with research productivity and higher quality [29]–[31].

The need for interdisciplinary collaboration has also been expressed for the current challenges to society derived from the relationships of environmental change to a range of crises, risks, and vulnerabilities. An uneven research capacity was highlighted with regard to the social sciences [32]. The fit of social scientists in funded multidisciplinary research teams, along with hierarchies in sciences, should be considered when following this path.

## Conclusions

Research on infections of poverty is too important to be neglected by researchers, in particular, social scientists. Their commitment to the deep-rooted causes of diseases—often surrounded by a web of circumstances and interplay of various factors—and the rationale for research on infectious diseases of poverty, bind their endeavors to the interests of the poor in a timely manner.

Social sciences research has played a significant role in research on NTDs in time and space, and still has more to contribute to this field. This could be accomplished with a substantial wealth of publications and the creation and/or participation in sustainable and effective community-directed interventions and health care programs adapted to hard-to-reach peoples and areas. Doing so would be better than succumbing to the penchant for generating and praising volumes of useless knowledge. The latter could appear impressive in terms of raw quantification of productivity; however, stating and re-stating the obvious, creates in the long run an imbalance instead of widening the breadth of investigative activities. This is likely not only doing a great disservice to the advancement of knowledge in the field, but also to the community, science, and policy interface.

In the continuation of this logic, conducting research and planning interventions for NTDs from the perspective of child poverty must receive further attention. Children are innocent, have lower coping capabilities (lower resistance to pathogens), and are in need of more protection. However, many are afflicted by the human, social, and economic misery exacerbated by recurrent and persistent armed conflicts and the related phenomenon of displaced populations, in which the spread of and increased vulnerability to some infectious diseases of poverty are embedded. Preferred military responses, a clear expression of human rights violations, are major moves forward enhancing child poverty and denying child development.

Risk reduction strategies and a wholesome preparedness are important to improve livelihoods, economies, and health, and this is why there is a need to promote a web style of research in tackling viciousness with the aim to impede or minimize vectors for prosperity. This would be by shielding human bodies against interrelated conditions, such as malnutrition linked to food insecurity, which itself depends on shocks on agricultural systems and their vulnerability. The handling of the latter is key in strengthening often-challenged human coping strategies and adaptation capacities themselves likely to lead to resilience crises, and so forth.

Research funding may not be a panacea, but like researchers from the natural, biomedical, and engineering sciences, social scientists need it. Therefore, as they compete to earn the coveted research grants on NTDs as principal investigators (PIs) or co-investigators (CoPIs), they should not be neglected. Are they at an unfair disadvantage in comparison to non-social scientists, or do they not try hard enough? How they blend in multidisciplinary teams also deserves some attention. Even though it could seem easier said than done, social scientists are encouraged to team up with other scientists (note 4 in Text S1) and catch the attention of diverse bodies of funding institutions.

## Supporting Information

Text S1 **Manuscript notes.**
(DOCX)

## References

[1] Mantilla B (2011) Invisible plagues, invisible voices: a critical discourse analysis of neglected tropical diseases. Soc Med 6: 118–127.

[2] World Health Organization (2012) Global report for research on infectious diseases of poverty. Geneva: WHO/TDR on behalf of the Special Programme for Research and Training in Tropical Diseases. Available: http://www.who.int/tdr/publications/global_report/en/. Accessed 15 May 2014

[3] Reidpath DD, Allotey P, Pokhrel S (2011) Social sciences research in neglected tropical diseases 3: Investment in social science research in neglected diseases of poverty: a case study of Bill and Melinda Gates Foundation. Health Res Policy Syst 9: 2. 21210999 10.1186/1478-4505-9-2 PMC3022559

[4] Yamey G (2002) The world's most neglected diseases. Ignored by the pharmaceutical industry and by public-private partnerships. BMJ 325 (7360): 352.

[5] Regnier M (2011) NTDs: A new handle on old problems. Wellcome News Available: http://blog.wellcome.ac.uk/2012/01/06/neglected-tropical-diseases-new-handle-old-problems/. Accessed 15 May 2014.

[6] Reidpath DD, Allotey P, Pokhrel S (2011) Social sciences research in neglected tropical diseases 2: A bibliographic analysis. Health Res Policy Syst 9: 1. 21210997 10.1186/1478-4505-9-1 PMC3024304

[7] Bastien JW (1998) The Kiss of Death: Chagas' Disease in the Americas. Salt Lake City: University of Utah Press.

[8] Brandling-Bennett D (1998) An anthropologist on Chagas' disease. The Lancet 352: 1865.

[9] MOST NUFFIC IK Unit (2003) Control of Chagas disease through a Cultural Context Model: Proyecto Britanico Cardenal Maurer in Sucre, Bolivia. Best practice on indigenous knowledge. Bolivia BP-II. 22. Paris: UNESCO.

[10] Briceño-León R (1990) La Casa Enferma: Sociología de la Enfermedad de Chagas. Caracas: Fondo Editorial Acta Cientifica Venezolana.

[11] Lewis (1968) The Culture of Poverty. In: Moynihan DP, editor. On Understanding Poverty: Perspectives From the Social Sciences. New York, NY: Basic Books. pp. 187–200.

[12] Murray C (1984) Losing Ground: American Social Policy, 1950–1980. New York, NY: Basic Books.

[13] Ryan W (1976) *Blaming the Victim*. New York: Vintage Books.

[14] Gunarathna A (2000) Social conditions in Sri Lanka accelerate spread of dengue fever. International Committee of the Fourth International (ICFI). Available: http://www.wsws.org/en/articles/2000/12/deng-d05.html. Accessed 17 May 2013

[15] Clemmons L, Amazigo UV, Bissek AC, Noma M, Oyene U, et al (2002) Gender issues in the community-directed treatment with ivermectin (CDTI) of the African Programme for Onchocerciasis Control (APOC). Anna Trop Med Parasit 96: 59–74. 10.1179/000349802125000655 12081252

[16] Heijnders ML (2004) The dynamics of stigma in leprosy. Int J Leprosy 72: 437–447. 10.1489/1544-581X(2004)72<437:TDOSIL>2.0.CO;2 15755198

[17] Vlassoff C, Weiss M, Ovuga EBL, Eneanya C, Nwel PT, et al (2000) Gender and the stigma of onchocercal skin disease in Africa on women: What do we know? J Biosoc Sci 26: 37–53. 10.1016/s0277-9536(99)00389-5 10741573

[18] Pepin J, Mpia B, Iloasebe M (2002) Trypanosoma brucei gambiense African trypanosomiasis: differences between men and women in severity of disease and response to treatment. Trans R Soc Trop Med Hyg 96: 421–426. 12497980 10.1016/s0035-9203(02)90380-9

[19] Braga C, Dourado I, Ximenes R, Miranda J, Alexander N (2005) Bancroftian filariasis in an endemic area of Brazil: Differences between genders during puberty. Rev Soc Bras Med Trop 38: 224–228. 15895172 10.1590/s0037-86822005000300003

[20] Weiss MG (2008) Stigma and the Social Burden of Neglected Tropical Diseases. PLoS Negl Trop Dis 2: e237 10.1371/journal.pntd.0000237 18478049 PMC2359851

[21] Weiss MG, Ramakrishna J (2001) Stigma and Global Health: Developing a Research Agenda. International Conference “Stigma Interventions and Research for International Health.” 5–7 September 2001. Available: http://www.stigmaconference.nih.gov/FinalWeissPaper.htm. Accessed 19 May 2014

[22] Kimbrough W, Saliba V, Dahab M, Haskew C, Checchi F (2012) The burden of tuberculosis in crisis-affected populations: a systematic review. Lancet Infect Dis 12: 950–965 10.1016/S1473-3099(12)70225-6 23174381

[23] Azoh J (2013) Crowded Refugee Camps, Empty Stomachs and Dehydrated Bodies: Public Health Opportunities to End Multiple Child Jeopardy in sub-Saharan Africa. In: Proceedings of the13^th^ World Congress of Public Health, organized by the World Federation of Public Health Associations (WFPHA), in collaboration with the Ethiopian Public Health Association in Addis Ababa, Ethiopia (23–37 April 2012). Pianoro (BO), Italy: Medimond.

[24] Yamey G, Hotez P, Roy WG (2007) Neglected Tropical Diseases. BMJ 335: 0.

[25] Thylefors B, Alleman M (2006) Towards the elimination of onchocerciasis. Ann Trop Med 100: 733–746. 10.1179/136485906X112202 17227651

[26] Simmons M, Tebeza-Mor N, Putnak R (2012) Advances in the development of vaccines for dengue fever. Vaccine (Auckl) 2: 1–14.

[27] Danko JR, Beckett CG, Porter KR (2011) Development of dengue DNA vaccines. Vaccine 29: 7821–7826. 10.1016/j.vaccine.2011.07.019 21777640

[28] Inhorn MC (1995) Medical anthropology and epidemiology: divergences or convergences? Soc Sci Med 40: 285–290. 7899941 10.1016/0277-9536(94)e0029-r

[29] Beaver DD (2004) Does collaborative research have greater epistemic authority? Scientometrics 60: 399–408.

[30] Nick H, Ban L, Kaufmann L, Loughnan S, Peters K (2008) What makes an article influential? Predicting impact in social and personality psychology journal. Scientometrics 76: 169–185.

[31] Abramo G, D'Angelo CA, Solazzi M (2011) The relationship between scientists' research performance and the degree of internationalization of their research. Scientometrics 86: 629–643.

[32] World Social Science Report (2013) Changing Global Environments. OECD. 10.1787/9789264203419

