# Supplementary material for: Social Sciences Research on Infectious Diseases of Poverty: Too Little and Too Late?
Source: PLoS Negl Trop Dis. 2014 Jun 12;8(6):e2803. doi: 10.1371/journal.pntd.0002803 (PMC4055465; doi:10.1371/journal.pntd.0002803)
Supplement: Text S1 — Manuscript notes. (DOCX) [file pntd.0002803.s001.docx]

PLOS NTDs Notes

**Note 1**

Malaria, tuberculosis, and HIV/AIDS known as the big three, represent a noticeable exception with the special research initiatives and mobilization of funds by international donors and foundations (e.g., The Bill & Melinda Gates Foundation; The Global Fund to fight Aids, Tuberculosis and Malaria (GFATM)). Malaria, however, is not yet under control in most parts of sub-Saharan Africa despite a wider distribution of insecticide-treated mosquito nets, and directing further attention to children access at the household level.

**Note 2**

A part of the reality, perhaps overlooked, is that many sick persons do not go to hospitals, or cannot even access a dispensary. They remain at home and rely on the use of alternative treatments consisting of medicinal herbs, prayers, and fake pharmaceutical drugs.

The latter, antibiotics for the most, are illegally made available at market stalls under hot and humid conditions in sealed containers, but not necessarily original. In addition, they do not really have active ingredients (e.g. talcum powder; sodium bicarbonate), and/or have passed their expiration date by far. On the other hand, some malaria patients simply remain without using any treatment at all. In both cases, the common points are that they are very poor and die at home.

**Note 3**

Collaborative research, valued by international funding agencies (National Science Foundation; Inter-American Institute for Global Change; World Health Organization; International Development Research Center...), is also more and more encouraged in the social sciences.

**Note 4**

Remote sensing applications, spectral imagery analyses, climatic characterizations, vectors’ dispersal and competence, housing, lifestyles are examples of the diversity of expertise to bring to research on NTDs.
